# Dr. Antonio Gabrini

**Published:** 1909-04-03

**Authors:** 


Dr. Antonio Gabrini, of Lugano, Switzerland, professor and physician, who died last November at the age of 94, left property reported to be of a total value of over £1,000,000 sterling, and property in the United Kingdom amounting to £4,235 5s. 1d. He left several bequests for charitable purposes, including 20,000f. to the municipality of Lugano for the erection of a house for the recuperation of indigent persons who are not ill.

